# Programmed cell death: immune regulatory mechanisms in periodontitis and rheumatoid arthritis

**DOI:** 10.3389/fcell.2026.1815945

**Published:** 2026-10-12

**Authors:** Hei Chan, XuFeng Chen, Jianwen Deng, Yuyan Mai

**Affiliations:** 1 The First Affiliated Hospital of Guangdong Pharmaceutical University, Guangdong, China; 2 Guangdong Provincial Hospital of Integrated Traditional Chinese and Western Medicine, Guangzhou/Foshan, China; 3 Medical Center of Stomatology, The First Affiliated Hospital, Jinan University, Guangzhou, China; 4 Zeren Dental Clinic (Guangzhou) Co., Ltd., Guangzhou, China

**Keywords:** apoptosis, immune, periodontitis, programmed cell death, rheumatoid arthritis

## Abstract

Periodontitis and rheumatoid arthritis, as chronic inflammatory diseases that share epidemiological associations and immunopathological characteristics, have various forms of programmed cell death (PCD) that play a core role in their pathogenesis, which form an important link between the two. The comorbidity of periodontitis and rheumatoid arthritis suggests that there might be a common immune regulatory mechanism, with PCD pathways playing a key role in regulating immune responses. Particularly, the Th17/Treg imbalance and apoptosis resistance in CD4^+^CD28^−^ T cells emerge as shared T cell–centered mechanisms bridging periodontal infection to rheumatoid autoimmunity. Studying how PCD works in periodontitis and rheumatoid arthritis can help uncover the potential links between the two diseases, which provides new targets for clinical treatment and improves patient prognosis. In this article, we’ll review relevant research and discuss how PCD is important for immune regulation in periodontitis and rheumatoid arthritis, which provides a theoretical basis for future treatment strategies for both conditions, helping to control the progression of inflammation more effectively.

## 1 Introduction

Programmed cell death (PCD) is a process through which cells undergo self-destruction via specific mechanisms, mainly including apoptosis, necroptosis, pyroptosis, and autophagy (Bursch et al., 2000). In immune regulation, PCD plays an important role by modulating the survival and function of immune cells, maintaining immune homeostasis. In recent years, an increasing body of research indicates that PCD plays a key role in the pathogenesis of various diseases, especially in chronic inflammatory diseases such as periodontitis (PD) and rheumatoid arthritis (RA) (Röhner et al., 2010).

Both PD and RA are chronic inflammatory diseases characterized by dysregulated local or systemic immune responses. Specifically, PD leads to inflammation and damage to the gums and alveolar bone (Ray, 2023), whereas RA, an autoimmune condition, involves chronic synovial inflammation that primarily affects the joints, resulting in the destruction of cartilage and bone (Fang et al., 2020). The chronic inflammation caused by PD may exacerbate RA’s pathological process by promoting the activation of immune cells and the release of cytokines (Cheng et al., 2024). Research has found that the shared pathophysiological features of these diseases are likely linked to the immune regulatory network related to PCD. Current studies show that the activation of PCD can induce inflammatory responses and bone resorption in osteoclasts, affecting the bone microenvironment (Lian et al., 2024). Dysregulated PCD may lead to an imbalance between pro- and anti-inflammatory actions, and between bone resorption and formation, thereby exacerbating the pathological processes of both PD and RA (Lin et al., 2022; Jiang et al., 2022). Therefore, exploring the connection between PCD in these two diseases, especially how it links the inflammatory bone destruction of PD and RA through the immune regulatory network, is crucial for understanding their underlying mechanisms and developing new treatment strategies.

## 2 Fundamental mechanisms of programmed cell death

PCD encompasses not only apoptosis but also autophagy, necroptosis, pyroptosis, and NETosis, each with distinct molecular machinery and immunological consequences.

### 2.1 Molecular mechanisms of apoptosis and autophagy

Apoptosis and autophagy are two evolutionarily conserved and finely regulated cellular fate determination programs that can either work against each other, cooperate, or even switch roles under stress.

Apoptosis is primarily initiated via the mitochondrial pathway (intrinsic): when triggered by signals such as oxidative stress or DNA damage, pro-apoptotic B-cell lymphoma-2 (Bcl-2) family proteins (specifically, Bcl-2-associated X protein [Bax] and Bcl-2 antagonist/killer [Bak]) oligomerize and increase mitochondrial outer membrane permeability, releasing cytochrome c. Cytochrome c subsequently binds to apoptotic protease-activating factor-1 (APAF-1) to form the apoptosome, which activates initiator caspase-9. Caspase-9 subsequently cleaves effector caspases-3/7, mediating cellular disintegration and forming apoptotic bodies (Antonsson, 2001; Roufayel, 2016). The extrinsic apoptotic pathway is initiated by the binding of death receptors—such as Fas (Fas cell surface death receptor; also known as CD95) or tumor necrosis factor receptor 1 (TNFR1)—to their respective ligands, Fas ligand (FasL) or tumor necrosis factor-α (TNF-α). Ligand binding recruits the adaptor protein Fas-associated death domain protein (FADD), which leads to the activation of caspase-8. Activated caspase-8 can either directly propagate the caspase cascade to execute apoptosis, or indirectly amplify the mitochondrial apoptotic signaling by cleaving the BH3-interacting domain death agonist (Bid) protein (Hehlgans and Pfeffer, 2005), which translocates to the mitochondria to trigger the intrinsic pathway.

Autophagy initiation depends on cellular energy and nutrient status and is mainly regulated by the AMP-activated protein kinase (AMPK)–mechanistic target of rapamycin complex 1 (mTORC1) axis. AMPK phosphorylates the Unc-51-like autophagy-activating kinase 1 (ULK1) complex, thereby alleviating mTORC1-mediated inhibition. This activates the Beclin 1 –class III phosphatidylinositol 3-kinase (Vps34)–autophagy-related protein 14 L (ATG14 L) core complex, which catalyzes the production of phosphatidylinositol 3-phosphate (PI3P) (Egan et al., 2011; Matsunaga et al., 2009). PI3P recruits downstream ATG systems (such as the ATG12–ATG5–ATG16L1 complex and microtubule-associated protein 1A/1B light chain 3 (LC3) lipidation) (Bomont, 2019; Iriondo et al., 2023; Zhang et al., 2024) to drive autophagosome formation, expansion, and fusion with lysosomes for degradation (Chen et al., 2012).

The two processes are dynamically cross-regulated via multiple molecular interfaces. Anti-apoptotic Bcl-2/B-cell lymphoma-extra large (Bcl-XL) binds and inhibits Beclin 1, thereby suppressing autophagy (Zhou et al., 2011); BH3-only proteins competitively disrupt this binding, promoting autophagy and delaying apoptosis. Activated caspases can cleave Beclin 1, altering the autophagic process (Luo and Rubinsztein, 2010; Mukhopadhyay et al., 2014), while excessive activation of autophagy can also convert to type II programmed cell death (Rami and Kögel, 2008). Therefore, key nodes such as the Bcl-2 family, Beclin 1, and caspases combine signals from their environment to decide the final fate of cells under stress.

### 2.2 Molecular mechanisms of necroptosis, pyroptosis, and NETosis

Necroptosis, pyroptosis, and NETosis are three forms of programmed cell death characterized by cell membrane rupture and strong inflammatory responses.

Necroptosis is mainly driven by the receptor-interacting protein kinase (RIPK) 1-RIPK3-mixed lineage kinase domain-like (MLKL) signaling axis. When death receptors (such as TNFR-1) are activated and caspase-8 activity is inhibited, RIPK1 phosphorylates RIPK3 to form a “necrosome,” which subsequently phosphorylates MLKL. The activated MLKL oligomerizes and moves to the cell membrane, disrupting its integrity and ultimately leading to cellular swelling and rupture (Someda et al., 2020; Ye et al., 2023). Pyroptosis is mediated by caspase-1 activated by the inflammasome (such as NOD-like receptor family pyrin domain containing 3, NLRP3) or human caspase-4/5/11 (caspase-11 in mice). These activated caspases cleave gasdermin D (GSDMD), releasing its N-terminal domain that creates pores in the cell membrane, causing ion influx, cell swelling, and membrane rupture, while also releasing pro-inflammatory factors such as interleukin (IL)-1β and IL-18 (Rathinam et al., 2019). NETosis is a unique form of cell death in neutrophils. It relies on nicotinamide adenine dinucleotide phosphate (NADPH) oxidase-generated reactive oxygen species (ROS), which activate peptidylarginine deiminase 4 (PAD4). PAD4 mediates extensive histone citrullination, resulting in chromatin decondensation. Subsequent breakdown of the nuclear and plasma membranes releases neutrophil extracellular traps (NETs) that can entrap pathogens but also exacerbate local tissue inflammation (Vorobjeva and Chernyak, 2020). Despite differing initiation mechanisms and key molecules, these three forms of cell death share common outcomes of irreversible plasma membrane rupture, release of intracellular danger signal molecules, and intense immune/inflammatory responses. They can collectively be referred to as “membrane rupture-type programmed cell death,” playing key roles in fighting infections and various chronic inflammatory diseases.

## 3 Interaction between immune dysregulation and programmed cell death in the context of periodontitis

The association between PCD and PD is well-established, particularly concerning key cell types within the periodontal microenvironment such as gingival fibroblasts, T cells, B cells, monocytes/macrophages, and neutrophils. Periodontal pathogens, exemplified by *Porphyromonas gingivalis* (*P. gingivalis*) can trigger host immune responses, leading to a substantial release of pro-inflammatory cytokines, thereby inducing PCD (including apoptosis, pyroptosis, and necroptosis).

### 3.1 Periodontitis and pyroptosis and necroptosis

Both pyroptosis and necroptosis are forms of inflammatory cell death. In addition to clearing associated pathogens, they can promote the secretion of inflammatory cytokines such as IL-1β and IL-18 (Li et al., 2021; Kitur et al., 2016; Liu et al., 2022; Ding et al., 2020). Pyroptosis is primarily mediated by the NLRP3 inflammasome activating caspase-1, which then cleaves GSDMD; necroptosis is mediated by RIPK-1 and RIPK-3, as well as MLKL. Periodontal pathogens like *P. gingivalis* and its cell wall components such as Lipopolysaccharide (LPS) can bind to Toll-like receptor (TLR) - 2/4 on host cells (Xu et al., 2022; Banavar et al., 2024), activating the NLRP3/caspase-1/GSDMD and RIPK1/3-MLKL pathways, thereby inducing pyroptosis and necroptosis (Li et al., 2021; Li et al., 2019; Huang et al., 2020; Ke et al., 2016; Geng et al., 2022; Li et al., 2018). Studies show that untreated PD patients have higher levels of RIPK1/3, MLKL, NLRP3, and caspase-1 in inflammatory gingival tissues compared to healthy controls (Li et al., 2021; Shi et al., 2019; Surlin et al., 2021), consistent with animal experimental studies (Li et al., 2021; Chen et al., 2021; Yamaguchi et al., 2017; Yue et al., 2024). After initial treatment, the expression levels of these markers were reduced (Bostanci et al., 2009). Furthermore, the administration of RIPK1/3, MLKL, NLRP3, and caspase-1 inhibitors in PD animal models was found to attenuate periodontal bone loss and suppress the expression of IL-1β and IL-18 (Bostanci et al., 2009; Li L. et al., 2023; Ye et al., 2024). These findings indicate that pyroptosis and necroptosis pathways play important roles in the pathogenesis of PD and may also be potential therapeutic targets for PD.

### 3.2 Periodontitis and apoptosis and autophagy

The apoptosis-related receptor FasL is expressed in many cell types, including gingival fibroblasts, B cells, T cells, and macrophages (Mishra et al., 2017; Truman et al., 1997; Lee et al., 2008). In healthy periodontal tissues, the expression of Fas, FasL, and anti-apoptotic Bcl-2 proteins is tightly controlled to maintain a balance between anti-apoptotic and pro-apoptotic effects in the periodontal microenvironment. Generally, activated T and B cells undergo apoptosis after antigen clearance to limit tissue damage caused by immune responses. In immunohistochemical studies and *in vitro* studies of periodontal pathogens, increased apoptosis levels have been observed (Trindade et al., 2012; Gamonal et al., 2001). Pro-apoptotic indicators such as Fas, FasL, and caspase-3 can be detected in gingival crevicular fluid, saliva, and periodontal tissues (Gamonal et al., 2001; Pradeep et al., 2016; Bantel et al., 2005; Dabiri et al., 2016), showing significant upregulation in populations of patients with PD and correlating positively with the severity of PD (Dabiri et al., 2016; Abuhussein et al., 2014). This phenomenon is closely linked to the periodontal pathogen *P. gingivalis*, whose virulence factors can regulate the level of apoptosis by recognizing TLR-2/4 and activating downstream signaling pathways such as nuclear factor-κB (NF-κB), mitogen-activated protein kinases (MAPKs), janus kinases/signal transducer and activator of transcription (JAK/STAT) and phosphatidylinositol-3-kinase/serine-threonine kinase (PI3K/Akt), thereby contributing to the pathological progression of periodontal tissues (Kang et al., 2019; Seo et al., 2012; Song et al., 2020; Yilmaz et al., 2004). It has been reported that apoptosis is closely related to earlier periodontal inflammation (Ebersole et al., 2021). *P. gingivalis* can modulate cell death in various cell types. Early work by O. Koulouri et al., revealed that apoptotic cells in inflamed periodontal tissues consist primarily of gingival fibroblasts, whereas the incidence of apoptosis in polymorphonuclear leukocytes, particularly neutrophils, is relatively low (Koulouri et al., 1999). In contrast to the delayed apoptosis phenomenon in monocytes/macrophages and neutrophils (Gamonal et al., 2003; Murray and Wilton, 2003), current research supports the induction of apoptosis by *P. gingivalis* in gingival fibroblasts (Wang et al., 1999; Imatani et al., 2004; Desta and Graves, 2007). Although *P. gingivalis* can initially upregulate the expression of the pro-apoptotic molecule Bax, under continuous stimulation by periodontal pathogens, the expression level of Bax decreases, while the expression of the anti-apoptotic molecule Bcl-2 increases (Nakhjiri et al., 2001). This may allow monocytes/macrophages and neutrophils recruited from relatively distant lymph nodes to the affected periodontal tissues to be less significantly affected by PCD. The delayed apoptosis of monocytes/macrophages and neutrophils may enable *P. gingivalis* to evade immune-related clearance and respond to pathogens by producing a large number of tissue-destructive factors, leading to more severe periodontal tissue destruction.

Increased autophagy can protect periodontal ligament stem cells or gingival fibroblasts from apoptosis (An et al., 2016; Bullon et al., 2012). In the inflammatory microenvironment of PD, LPS produced by *P. gingivalis* activates autophagy through the recognition of TLR-2 or TLR-4, thereby activating the NF-κB, JAK/STAT, and PI3K/AKT signaling pathways (Liu et al., 2018; Hagio-Izaki et al., 2018; Mo et al., 2019; Feng et al., 2024; Ma et al., 2021). *In vitro*, *P. gingivalis* and its LPS induce autophagy in human gingival fibroblasts and periodontal ligament cells, characterized by the formation of autophagosomes and the conversion of LC3-I to LC3-II (Bullon et al., 2012; Kim et al., 2022). S. Memmert et al. found that autophagy-related genes are expressed at higher levels in periodontal cells and tissues under inflammatory and infectious conditions, indicating that autophagy plays a key role in PD (Memmert et al., 2018). Moreover, various pro-inflammatory cytokines, such as TNF-α, IL-1β, and IL-6, may be suppressed by *P. gingivalis*-induced autophagy (Liu et al., 2018). However, Bullon et al. found that inhibiting autophagy with 3-methyladenine (3 MA) reduced the LC3-II/LC3-I ratio, increased intracellular *P. gingivalis*, and significantly elevated the proportion of apoptotic cells and the secretion of pro-inflammatory cytokines IL-1β, TNF-α, and IL-6 (Bullon et al., 2012; Liu et al., 2018). These results suggest that autophagy plays a protective role in PD.

When bacterial stimulation is excessive or persistent, autophagy can trigger apoptosis, leading to tissue destruction. Contrary to previous research conclusions, some studies suggest that autophagy participates in the pathological progression of PD and can positively regulate the production of inflammatory molecules (Kim et al., 2022). *P. gingivalis* can evade autophagic antibacterial effects by obstructing the fusion of autophagosomes with lysosomes (Liu M. et al., 2023). Gingipain-activated AKT/the mammalian target of rapamycin (mTOR) signaling inhibits the autophagic process of immune cells, promoting the survival of intracellular *P. gingivalis* (Meghil et al., 2019). Activation of the Akt/mTOR axis downregulates the formation of autophagosomes and the conversion of LC3-I to LC3-II. Furthermore, *P. gingivalis* can promote its long-term survival by enhancing Akt phosphorylation to inhibit the expression of pro-apoptotic Bad, Bim, and Bax proteins, and upregulate the expression of anti-apoptotic Bcl2 proteins, thus facilitating its colonization and replication in periodontal tissues (Pugazhenthi et al., 2000; Chen et al., 2007). Therefore, the effects triggered by autophagy or apoptosis may have destructive or protective functions in different disease contexts. While most studies support a protective role, others suggest that autophagy may contribute to pathological progression by enabling *P. gingivalis* to evade intracellular killing. These discrepancies may arise from differences in experimental models, infection duration, or bacterial strains, highlighting the need for standardized experimental frameworks to delineate the context-dependent functions of autophagy.

## 4 Interaction between immune dysregulation and programmed cell death in the context of rheumatoid arthritis

### 4.1 Rheumatoid arthritis and apoptosis and autophagy

The imbalance in the regulation of pro-apoptotic and anti-apoptotic processes in key cells such as synovial fibroblasts (FLS), macrophages, and lymphocytes in RA is considered a critical mechanism leading to progressive joint destruction. A deeper exploration of its core pathological links reveals that the dysfunction of the Fas/FasL apoptotic pathway plays a central role. Although synovial cells in RA can theoretically trigger apoptosis through the Fas/FasL pathway (Mosler et al., 2019; Okamoto et al., 1998; Kobayashi et al., 2000), the key pro-apoptotic ligand FasL is expressed at significantly low levels in synovial tissue (Asahara et al., 1997), which likely impairs apoptotic signaling and leads to resistance to apoptosis in synovial cells (especially fibroblasts and lymphocytes), thereby promoting abnormal growth and persistence of synovial tissue. This view is strongly supported by RA animal models: ectopic expression of FasL in these models effectively induces apoptosis in synovial cells and significantly improves joint inflammation, directly underscoring the therapeutic potential of restoring Fas/FasL pathway function (Liu and Pope, 2003). The abnormal elevation of the soluble form of Fas (sFas) further exacerbates dysfunction within the Fas/FasL pathway. Studies have found that while sFas has no intrinsic biological activity, it can bind FasL with high affinity (Hong et al., 2015). Compared to healthy controls, the level of sFas in the synovial fluid of RA patients is significantly elevated (Matsuno et al., 2001), which blocks FasL from binding to its membrane receptor Fas, thereby inhibiting the Fas-mediated apoptotic process. More critically, the RA synovial tissue microenvironment is characterized not only by elevated sFas levels but also by the persistent presence of pro-inflammatory cytokines, such as TNF-α and IL-1. Studies demonstrate that Fas, particularly in the context of elevated sFas, synergizes with these inflammatory factors to establish a robust anti-apoptotic network. The main mechanism is that these factors work together to regulate key pro-survival signaling pathways such as PI3K/AKT, NF-κB, and JAK/STAT (Wakisaka et al., 1998; Zhai et al., 2016; Zhao et al., 2021; Liu S. et al., 2019; Ji et al., 2021). Activation of these pathways directly drives the transcriptional upregulation of anti-apoptotic proteins such as Bcl-2, thereby significantly enhancing the resistance of synovial cells to apoptosis. *In vitro* experiments have demonstrated that blocking these pro-survival pathways with proteasome inhibitors (e.g., NF-κB inhibitors) effectively reverses the resistance of RA FLS to Fas-mediated apoptosis (Shi et al., 2016). These findings provide crucial evidence for developing novel RA therapeutic strategies targeting resistance to apoptosis and autophagy.

This apoptosis resistance is closely linked to elevated levels of autophagy in synovial tissue (Xu et al., 2013). Accumulating evidence indicates that autophagy-related markers (such as LC3, Beclin1, ATG5) are significantly increased in the synovial tissue of RA patients (Zhu et al., 2017). Pro-inflammatory factors (TNF-α, IL-1, IL-6) not only upregulate anti-apoptotic proteins (such as Bcl-2) through the same PI3K/AKT, NF-κB, JAK/STAT signaling pathways but also promote the expression of autophagy-related proteins (such as Beclin1) (Li et al., 2017; Wang and Gao, 2021; Hou and Gao, 2022). Current literature suggests that the increase in apoptosis resistance in RA synovium is significantly associated with the upregulation of autophagy, both of which are driven by the high local expression of inflammatory factors (TNFα, IL-1, IL-6) and their downstream key signaling pathways (PI3K/AKT, NF-κB, JAK/STAT). This combined effect (inhibiting apoptosis and promoting autophagy) provides a strong survival advantage for synovial cells, leading to persistent proliferation and difficulty in resolving inflammation, thereby establishing the core pathological basis for the progressive destruction of joints in RA. Despite the prevailing view that autophagy promotes synovial fibroblast survival, some evidence suggests that excessive autophagy can lead to cell death. The precise threshold at which autophagy transitions from a pro-survival to a pro-death mechanism in the RA synovium remains unclear, representing a critical gap in our understanding.

### 4.2 Rheumatoid arthritis and necroptosis, pyroptosis, and NETosis

In the study of RA, compared to apoptosis and autophagy, forms of highly regulated pro-inflammatory cell death such as necroptosis, pyroptosis, and NETosis have received relatively less attention, but they are increasingly recognized as important mechanisms driving the inflammatory cascade of the disease. Studies indicate that key mediators of necroptosis (such as RIPK1, RIPK3, and MLKL) and pyroptosis (such as the NLRP3 inflammasome, caspase-1, and GSDMD) are significantly elevated in the tissues of RA patients and RA mouse models (Liu L-M. et al., 2023; Wang et al., 2023; Xie et al., 2018; Choulaki et al., 2015; Liu Y. et al., 2019). Once these cell death programs are activated, they can exacerbate inflammation in the synovium, cause tissue damage, and precipitate systemic complications by releasing substantial quantities of potent pro-inflammatory factors (such as IL-1β, TNF-α, IL-18) and damage-associated molecular patterns (DAMPs) (Liu L-M. et al., 2023; Wu et al., 2022; Wu et al., 2020). *In vitro* and animal model studies provide strong evidence that the administration of specific bioactive compounds or small-molecule inhibitors targeting the necroptosis pathway (including RIPK1/RIPK3 or MLKL) or the pyroptosis pathway (including NLRP3, caspase-1, or GSDMD) can effectively reduce joint inflammation and damage in mouse models of RA (Jhun et al., 2019; Wu et al., 2024; Xu et al., 2021; Ma et al., 2024). These findings underscore that necroptosis and pyroptosis significantly contribute to the establishment and perpetuation of the pro-inflammatory microenvironment in RA.

NETs formation by neutrophils is a unique way neutrophils can die, called NETosis. In RA, both locally infiltrated neutrophils in the synovium and neutrophils in peripheral circulation show significantly enhanced NETosis activity. Notably, these elevated levels of NETosis are closely linked to the titer of a specific autoantibody in RA—anti-citrullinated protein antibodies (ACPAs) (Ma et al., 2024; Corsiero et al., 2016; Wright et al., 2021). An increasing number of studies support that NETosis is an important source of autoantigens in RA, particularly citrullinated antigens. The modified proteins (such as citrullinated histones) and antimicrobial peptides released by NETs are believed to play a key role in breaking the immune tolerance to self-antigens. Based on the role of NETosis, autoreactive B cells are activated, proliferated, and differentiated into plasma cells, producing a large amount of ACPAs. (Konig and Andrade, 2016; Zhang et al., 2020; Wright et al., 2014). These ACPAs not only serve as highly specific serological markers for RA. They also help maintain and amplify the inflammatory environment inside and outside the joints by forming immune complexes, activating complement, and stimulating macrophages, leading to a vicious cycle. In summary, necroptosis, pyroptosis, and NETosis, as three forms of regulated cell death that are highly pro-inflammatory, together constitute an indispensable link in the network of inflammation and tissue destruction in RA.

## 5 Bidirectional promotion mechanism of programmed cell death

### 5.1 Interaction between periodontitis and rheumatoid arthritis

The epidemiological association between PD and RA has been well-documented, with patients suffering from one disease showing an increased risk of developing the other. The immune cross-reaction between PD and RA represents a complex pathological association, with its core mechanism involving widespread immune issues triggered by periodontal pathogens such as *P. gingivalis*. The detection of oral bacterial DNA, including *P. gingivalis*, in the synovial fluid and serum of patients with active RA suggests that the pathogen or its components might break through local barriers and disseminate systemically (Moen et al., 2006). These pathogens not only cause local inflammation in periodontal tissues but have also been shown to induce the production of ACPAs (Li et al., 2016; Yang et al., 2024), which are specific serological marker for RA and may serve as a key link between the autoimmune responses of both diseases. Meanwhile, elevated pro-inflammatory factors (such as TNF-α, IL-1β, IL-6) in PD patients are released into systemic circulation, spreading inflammation throughout the body and significantly increasing susceptibility to RA (Kaur et al., 2012). The detailed molecular mechanisms by which these inflammatory signals engage PCD pathways are further discussed in Section 5.2.

At a deeper pathological level, the disruption of PCD is a major contributor to bone damage seen in both PD and RA (Bao et al., 2024; Adán et al., 2013). Chronic inflammation in the periodontal and synovial microenvironments creates a state of oxidative stress and sustained release of pro-inflammatory cytokines, which together impair bone homeostasis through multiple mechanisms (Wang et al., 2025; Zhou and Graves, 2022). On one hand, these inflammatory signals can directly compromise osteoblast survival and function, weakening bone formation (Zhou and Graves, 2022); on the other hand, they promote osteoclast differentiation and activity, driving pathological bone resorption via the nuclear factor-κB ligand (RANKL)/NF-κB axis (Gravallese and Schett, 2018; Crotti et al., 2015). Critically, there exists a self-perpetuating cycle whereby PCD activation amplifies local inflammation, which in turn further stimulates PCD and bone destruction, forming a vicious loop of “inflammation → PCD activation → bone resorption ↑/bone formation ↓ → sustained inflammation” that ultimately drives alveolar bone loss in PD and joint erosion in RA.

### 5.2 Integrated mechanistic model of PCD pathway cross-talk in periodontitis and rheumatoid arthritis

The pathological overlap between PD and RA is not merely epidemiological but is rooted in a shared molecular architecture wherein distinct PCD pathways do not operate in isolation but engage in dynamic, context-dependent cross-talk. This section presents an integrated mechanistic model that delineates how the convergence and divergence of apoptosis, autophagy, necroptosis, pyroptosis, and NETosis create a self-perpetuating inflammatory cycle in both diseases, ultimately converging on bone destruction (Figure 1).

**FIGURE 1:**
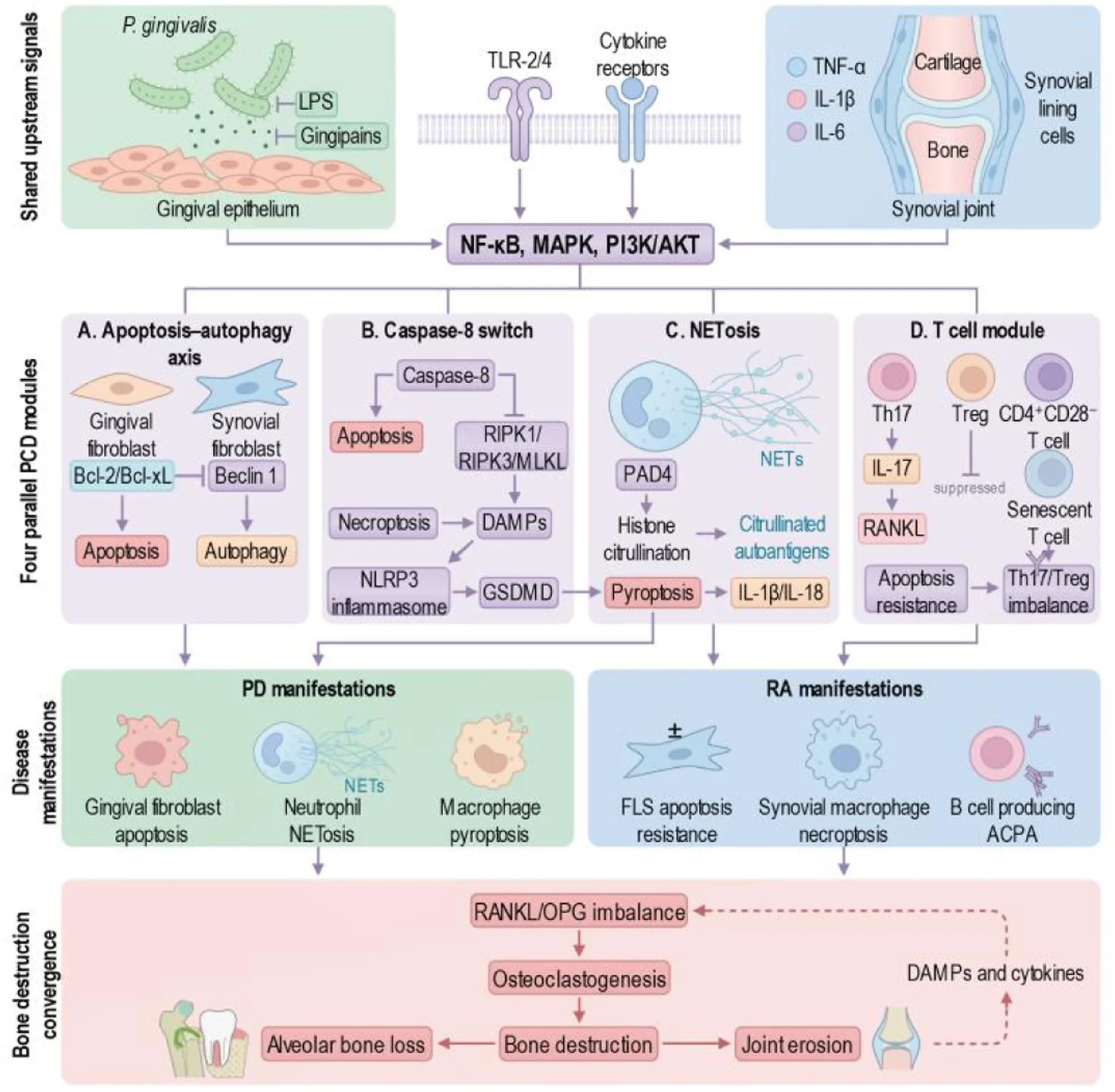
Integrated mechanistic model of programmed cell death (PCD) pathway cross-talk in periodontitis (PD) and rheumatoid arthritis (RA). (By Figdraw). Periodontal pathogens (*P. gingivalis*) and pro-inflammatory cytokines (TNF-α, IL-1β, IL-6) activate TLR-2/4 and downstream NF-κB, MAPK, and PI3K/AKT signaling hubs, which simultaneously prime four core PCD modules: **(A)** the apoptosis–autophagy axis regulated by Bcl-2/Beclin 1 cross-talk; **(B)** the caspase-8 switch that determines cell fate between apoptosis and RIPK1/RIPK3/MLKL-mediated necroptosis, with MLKL pores activating the NLRP3 inflammasome and GSDMD-dependent pyroptosis, leading to IL-1β/IL-18 release; **(C)** NETosis, driven by PAD4-mediated histone citrullination and NET release; and **(D)** the T cell module, in which Th17/Treg imbalance, CD4⁺CD28⁻ T cell apoptosis resistance, and T cell senescence converge on IL-17 and RANKL production. In PD, these pathways manifest as gingival fibroblast apoptosis, neutrophil NETosis, and macrophage pyroptosis; in RA, they manifest as FLS apoptosis resistance, synovial macrophage necroptosis, and ACPA production. All pathways converge on RANKL/OPG axis imbalance, driving osteoclastogenesis and bone destruction—alveolar bone loss in PD and joint erosion in RA. Positive feedback loops from tissue damage amplify upstream inflammation, perpetuating the vicious cycle. Solid arrows indicate activation; T-bar lines indicate inhibition; dashed lines indicate regulatory cross-talk. ACPA, anti-citrullinated protein antibody; FLS, fibroblast-like synoviocyte; GSDMD, gasdermin D; MLKL, mixed lineage kinase domain-like; NET, neutrophil extracellular trap; NLRP3, NOD-like receptor family pyrin domain containing 3; OPG, osteoprotegerin; PAD4, peptidylarginine deiminase 4; RANKL, receptor activator of nuclear factor-κB ligand; RIPK, receptor-interacting protein kinase.

#### 5.2.1 Upstream convergence: microbial and inflammatory signals as shared initiators

In PD, the dysbiotic subgingival microbiota, particularly *P. gingivalis* and its virulence factors (e.g., LPS, gingipains), activates host pattern recognition receptors (PRRs), primarily TLR-2/4, on gingival fibroblasts, macrophages, and neutrophils (Polishchuk et al., 2025). This recognition triggers NF-κB and MAPK signaling cascades, which simultaneously prime multiple PCD pathways (Xu et al., 2022). In RA, although the initiating trigger is autoimmune rather than infectious, the downstream signaling landscape is remarkably similar: pro-inflammatory cytokines—TNF-α, IL-1β and IL-6—engage their respective receptors and converge on the same NF-κB, MAPK, and PI3K/Akt hubs (Zhao et al., 2021; Shahbaz et al., 2024). Thus, both diseases share a common upstream signaling topology that licenses the activation of diverse PCD modalities.

#### 5.2.2 The apoptosis-autophagy axis: a double-edged sword in synovial and gingival tissues

A critical node of PCD cross-talk lies in the reciprocal regulation between apoptosis and autophagy, mediated by the Bcl-2 family and Beclin 1. In healthy tissues, anti-apoptotic Bcl-2 and Bcl-xL bind Beclin 1 via its BH3 domain, thereby suppressing autophagy initiation. Under inflammatory stress, however, BH3-only proteins (e.g., Bim, Bad) competitively disrupt this interaction, liberating Beclin 1 to activate Vps34 complex and drive autophagosome formation (Levine and Kroemer, 2008; Li YQ. et al., 2023).

In RA, this axis is pathologically skewed toward survival. FLS exhibit marked resistance to apoptosis, a phenomenon intimately linked to enhanced autophagic flux. TNF-α, abundantly present in RA synovium, upregulates autophagy-related proteins (Beclin 1, LC3-II, ATG5) in FLS (Huang et al., 2026), which in turn antagonizes TNF-α-induced apoptosis. Experimental evidence demonstrates that pharmacological inhibition of autophagy (e.g., with 3-methyladenine or chloroquine) restores the sensitivity of RA-FLS to apoptotic stimuli, underscoring autophagy as a pro-survival mechanism that sustains synovial hyperplasia (Kato et al., 2014; Lin et al., 2013). However, this relationship is not unidirectional; under severe endoplasmic reticulum stress, autophagy can transition to a pro-death function (type II PCD), highlighting the context-dependent duality of this pathway (Djavaheri-Mergny et al., 2006).

In PD, the apoptosis-autophagy balance is similarly disrupted but exhibits disease-stage dependency. During early infection, *P. gingivalis* LPS induces autophagy in gingival fibroblasts and periodontal ligament cells (Oka and Li, 2021), which functions protectively by limiting intracellular bacterial survival and suppressing apoptosis. Conversely, sustained bacterial stimulation or high-multiplicity infection triggers a shift wherein autophagy facilitates apoptosis, leading to tissue destruction (Bullon et al., 2012). Notably, *P. gingivalis* can subvert autophagy by blocking autophagosome-lysosome fusion, thereby exploiting the pathway for intracellular persistence (Kang et al., 2025). This bacterial evasion strategy represents a critical divergence point between PD and RA: while RA autophagy is primarily host-driven, PD autophagy is actively manipulated by periodontal pathogens.

#### 5.2.3 Caspase-8 as the molecular switch between apoptosis and necroptosis

Caspase-8 serves as a pivotal decision node that determines whether a cell undergoes apoptotic or necroptotic death. In the extrinsic apoptotic pathway, caspase-8 activation triggers the caspase cascade (caspase-3/7) leading to controlled cell demise. Crucially, active caspase-8 also cleaves and inactivates RIPK1 and RIPK3, thereby preventing necroptosis (Amaral and Bortoluci, 2020). When caspase-8 activity is compromised or blocked, the cellular fate shifts from apoptosis toward RIPK1/RIPK3/MLKL-mediated necroptosis, unleashing potent inflammatory responses.

In PD, *P. gingivalis* infection activates the caspase-8 cascade in macrophages as part of Z-DNA binding protein 1 (Zbp1)-mediated PANoptosis, wherein cleaved caspase-8 cooperates with RIPK3 and MLKL to drive inflammatory cell death (Wu et al., 2025). Importantly, this caspase-8 activation occurs concurrently with, rather than in opposition to, necroptosis signaling, representing a distinct feature of PANoptotic cell death in periodontal inflammation. This pathological shift is pathologically significant because necroptosis, unlike apoptosis, culminates in plasma membrane rupture and the release of DAMPs—such as high-mobility group box 1 (HMGB1), ATP, and mitochondrial DNA—which amplify local inflammation and recruit additional immune cells (Zhang K. et al., 2023; Seo et al., 2021). Elevated expression of RIPK1, RIPK3, and MLKL has been documented in inflamed gingival tissues from periodontitis patients, and pharmacological inhibition of RIPK3 or MLKL attenuates alveolar bone loss in experimental models (Shi et al., 2019).

In RA, the necroptosis pathway is activated in synovial macrophages and FLS, driven by chronic TNF-α signaling. Under physiological conditions, caspase-8 acts as a brake on necroptosis by cleaving RIPK1 and RIPK3; however, in the RA inflammatory microenvironment, the balance is tilted toward necroptosis due to sustained TNF-α-driven RIPK1/RIPK3 activation and the dominant pro-survival signaling that suppresses caspase-8-mediated apoptosis (Trimova et al., 2020; Zheng et al., 2025). The resulting DAMP release fuels a feed-forward inflammatory loop: HMGB1 and ATP activate the NLRP3 inflammasome in neighboring macrophages and dendritic cells, promoting IL-1β and IL-18 maturation and further exacerbating synovitis (Ludes et al., 2021).

#### 5.2.4 Necroptosis-pyroptosis convergence: DAMP-mediated inflammasome activation

The cross-talk between necroptosis and pyroptosis is mediated by the release of DAMPs from necroptotic cells, which act as endogenous adjuvants to trigger inflammasome assembly. In the canonical pyroptosis pathway, the NLRP3 inflammasome activates caspase-1, which cleaves GSDMD to form membrane pores and drives the secretion of mature IL-1β and IL-18. In non-canonical pyroptosis, caspase-4/5 (human) or caspase-11 (mouse) directly recognizes LPS and cleaves GSDMD (Exconde et al., 2023).

In PD, *P. gingivalis* LPS is a potent activator of both canonical and non-canonical pyroptosis in gingival fibroblasts, macrophages, and periodontal ligament cells. The resulting IL-1β upregulates receptor activator of RANKL expression in osteoblasts and fibroblasts, directly promoting osteoclastogenesis and alveolar bone resorption (Huang et al., 2025). Simultaneously, pyroptotic cell death releases additional DAMPs, creating a self-sustaining cycle of inflammation and bone destruction.

In RA, pyroptosis is similarly driven by NLRP3 inflammasome activation in FLS, CD4^+^ T cells, and macrophages, with elevated levels of caspase-1, GSDMD, and IL-1β in RA synovial tissues (Zhang X. et al., 2023). The IL-1β produced not only drives local inflammation but also systemically amplifies the acute-phase response. Importantly, the necroptosis executioner MLKL has been shown to directly activate the NLRP3 inflammasome independent of cell lysis, providing a mechanistic link between these two lytic PCD pathways (Lei et al., 2023). This cross-talk is particularly relevant in the shared bone destruction phenotype of PD and RA, as both pathways converge on RANKL upregulation and osteoclast activation.

#### 5.2.5 NETosis: the immunological bridge linking periodontal infection to autoimmunity

NETosis represents a unique form of PCD that bridges the infectious etiology of PD and the autoimmune pathogenesis of RA. In PD, neutrophils undergo NETosis in response to *P. gingivalis*, *Aggregatibacter actinomycetemcomitans* (*A. actinomycetemcomitans*), and their products (LPS, leukotoxins) (Shahbaz et al., 2024; Konig et al., 2016). This process is critically dependent on peptidylarginine deiminase (PAD) activation, which citrullinates histones and other proteins, facilitating chromatin decondensation and NETs release (Spengler et al., 2015).

The citrullinated proteins contained within NETs—particularly citrullinated histones (H2A, H3, H4), α-enolase, and vimentin—serve as potent autoantigens (Roe, 2025). In genetically susceptible individuals (e.g., those carrying HLA-DRB1 shared epitope alleles or PADI4 polymorphisms), these citrullinated antigens breach immune tolerance and drive the production of ACPAs, a hallmark of RA (Scally et al., 2013). This establishes a direct molecular link: periodontal NETosis generates the autoantigens that initiate or amplify RA-specific autoimmunity.

Conversely, the systemic inflammation of RA (characterized by circulating TNF-α, IL-1β, and IL-17A) enhances NETosis in peripheral neutrophils (Sun et al., 2025). These neutrophils, once recruited to periodontal tissues, release NETs enriched in proteases (neutrophil elastase, cathepsin G) and citrullinated proteins, which directly degrade periodontal connective tissue and amplify local inflammation (Shahbaz et al., 2024). Thus, NETosis creates a bidirectional vicious cycle: PD-driven NETosis generates citrullinated autoantigens that promote RA, while RA-driven systemic inflammation enhances periodontal NETosis, exacerbating tissue destruction.

#### 5.2.6 Convergence on bone destruction: the RANKL/OPG axis as the final common pathway

Regardless of the specific PCD modality activated, the integrated PCD network in both PD and RA converges on dysregulated bone remodeling through the RANKL/osteoprotegerin (OPG) axis (Haynes and Crotti, 2003). Inflammatory cytokines (TNF-α, IL-1β, IL-6) and DAMPs released from necroptotic, pyroptotic, and NETotic cells upregulate RANKL expression while suppressing OPG, tilting the balance toward osteoclastogenesis (Kang et al., 2021; Alippe et al., 2017).

In PD, this results in alveolar bone loss and clinical attachment destruction; in RA, it manifests as marginal bone erosion and joint deformity (Panezai et al., 2018). Notably, the comorbidity of PD and RA is associated with the highest serum OPG levels and most severe bone destruction, suggesting that the simultaneous activation of multiple PCD pathways in both diseases creates a synergistic, rather than merely additive, effect on bone resorption (Xu et al., 2022). The distinct roles of each PCD pathway in PD and RA are summarized in Table 1.

**TABLE 1:** Comparative roles of programmed cell death pathways in periodontitis and rheumatoid arthritis.

PCD Pathway	Role in Periodontitis (PD)	Role in Rheumatoid Arthritis (RA)
Apoptosis	Induced in gingival fibroblasts; delayed in neutrophils/macrophages, facilitating pathogen persistence	Resistance in synovial fibroblasts (FLS) via Bcl-2/Mcl-1 upregulation; drives synovial hyperplasia
Autophagy	Protective via pathogen clearance; excessive levels may trigger apoptosis; manipulated by P. gingivalis for intracellular persistence	Elevated in synovium; promotes FLS survival and apoptosis resistance; dual role under ER stress
Necroptosis	RIPK1/3-MLKL mediated; elevated in inflamed gingiva; correlates with disease severity	RIPK1/RIPK3/MLKL elevated; drives synovial inflammation and joint erosion
Pyroptosis	NLRP3-Caspase-1-GSDMD mediated; releases IL-1β/IL-18; amplifies local inflammation	NLRP3 inflammasome activation in FLS, macrophages, and T cells; contributes to synovitis and bone erosion
NETosis	Triggered by P. gingivalis and A. actinomycetemcomitans; releases citrullinated autoantigens	Primary source of citrullinated autoantigens; drives ACPA production; fuels synovial inflammation

Abbreviations: ACPA, anti-citrullinated protein antibody; ER, endoplasmic reticulum; FLS, fibroblast-like synoviocyte; GSDMD, gasdermin D; IL, interleukin; MLKL, mixed lineage kinase domain-like; NET, neutrophil extracellular trap; NLRP3, NOD-like receptor family pyrin domain containing 3; PCD, programmed cell death; RIPK, receptor-interacting protein kinase.

#### 5.2.7 T cell subsets and apoptosis: Th17/Treg imbalance as a shared pathological nexus

T cells, particularly CD4^+^ helper subsets, constitute a critical nexus linking PCD dysregulation to the shared bone-destructive phenotype of PD and RA. The Th17/Treg axis is the most mechanistically informative node. Both diseases feature a pathological shift toward Th17 dominance with Treg functional compromise. In PD, *P. gingivalis* promotes Th17 differentiation while impairing Treg suppressive capacity, and IL-17 produced by Th17 cells upregulates RANKL in fibroblasts and osteoblasts, directly driving osteoclastogenesis and alveolar bone resorption (Bi et al., 2019). In RA, Th17 cells accumulate in the synovium and amplify inflammation and joint erosion, whereas Tregs in synovial fluid lose suppressive function (Noack and Miossec, 2014). This shared imbalance suggests a common immunological link underlying the epidemiological association between the two diseases.

A central mechanism perpetuating this imbalance is the differential susceptibility of T cell subsets to apoptosis, which is best characterized in RA. Despite synovial T cells displaying an apoptosis-prone phenotype, apoptosis is not detectable in synovial fluid; these cells rapidly undergo spontaneous apoptosis upon isolation from the joint, indicating that the synovial microenvironment actively rescues them from death (Salmon et al., 1997). Synovial fibroblasts confer this resistance through integrin–ligand interactions that upregulate Bcl-xL, maintaining a Bcl-xL^high/Bcl-2^low phenotype resistant to apoptotic stimuli (Salmon et al., 1997). Particularly pathogenic are CD4^+^CD28^−^ T cells, which are clonally expanded, autoreactive, and resistant to apoptosis upon IL-2 withdrawal, with elevated Bcl-2 expression and defective Fas signaling (Schirmer et al., 1998). These apoptosis-resistant cells perpetuate the Th17/Treg imbalance and contribute to RA chronicity.

In PD, by contrast, T cell apoptosis is more context-dependent and shaped by both bacterial factors and the local microenvironment. The *P. gingivalis* HmuY protein upregulates Bcl-2 in CD3^+^ T cells from chronic periodontitis patients, which may delay T cell apoptosis and modulate immune clearance, thereby favoring pathogen persistence (Carvalho-Filho et al., 2013). Conversely, *P. gingivalis*-specific T cell lines undergo activation-induced apoptosis after antigen restimulation *in vitro* (Gemmell et al., 1999). *In situ*, however, lymphocytes in periodontal lesions express Fas but lack sufficient FasL, indicating that although they are susceptible to Fas-mediated apoptosis, the death signal is not efficiently delivered within the lesion (Sawa et al., 1999). These observations are not contradictory; they reflect differences in stimulation mode, T cell subset, and death-ligand availability. Such context-dependent regulation may allow *P. gingivalis* to evade immune surveillance while sustaining a permissive inflammatory environment, contributing to Th17/Treg imbalance.

Beyond apoptosis, T cell senescence has emerged as an additional mechanism linking PD to RA. Ligature-induced periodontitis in mice accelerates CD4^+^ T cell senescence, generating a “senescence-primed” status that may enter systemic circulation and exacerbate experimental arthritis (Li et al., 2026). Senescent CD4^+^CD28^−^ T cells exhibit a Th17-biased phenotype, are resistant to apoptosis, and can themselves express RANKL, thereby amplifying osteoclastogenic signaling (González-Osuna et al., 2022). This positions PD-induced T cell senescence as a potentially important mechanism bridging periodontal infection to RA exacerbation.

### 5.3 Sex-specific and genetic factors

Sex dimorphism modulates PCD pathways in both PD and RA. In periodontal tissues, females exhibit higher gingival apoptotic cell counts than males, with potential implications for inflammatory resolution and tissue repair (Johnson and Wikle, 2014). In RA, the female-to-male ratio of approximately 3:1 suggests that sex hormones—particularly estrogen and testosterone—may influence PCD regulation, though the underlying mechanisms remain incompletely understood (Raine and Giles, 2022).

At the genetic level, transcriptomic analyses have identified shared cross-talk genes between PD and RA, including TIMP1, LGALS1, GPX4, VOPP1, and AIM2, many of which are linked to pyroptosis pathways (Jing et al., 2021). Bioinformatics approaches have further revealed significant molecular overlaps between PD and RA, encompassing dysregulation of inflammatory mediators, immune response pathways, oxidative stress, and extracellular matrix remodeling (Martínez-García and Hernández-Lemus, 2024). Furthermore, the PADI4 GTG haplotype has been associated with increased NETosis in RA patients with periodontitis, with circulating neutrophil NET release influenced by this haplotype and associated with both diseases (Oliveira et al., 2024).

These genetic links provide a molecular basis for the comorbidity of PD and RA and offer potential biomarkers and therapeutic targets. However, most genetic association studies are observational and lack functional validation, underscoring the need for mechanistic investigations to determine whether these variants causally influence PCD regulation or represent secondary epiphenomena.

## 6 Clinical evidence and translational limitations

Despite robust preclinical evidence, human clinical data validating PCD as a therapeutic target in PD and RA remain scarce. Most conclusions derive from *in vitro* studies and animal models, which fail to fully replicate the chronic, heterogeneous nature of human disease.

Key translational barriers include: (i) the lack of validated, non-invasive biomarkers for specific PCD pathways; (ii) the context-dependent dual roles of pathways such as autophagy, raising off-target concerns; and (iii) the limited clinical evaluation of PCD-targeting agents. For example, the RIPK1 inhibitor GSK2982772 showed no significant benefit over placebo in a Phase II RA trial (Weisel et al., 2021), and no NLRP3 inhibitor has yet been approved for either disease. Targeting apoptosis resistance in pathogenic T cell subsets may offer a shared therapeutic strategy, but requires pathway-selective modulation.

Future efforts should prioritize biomarker-stratified clinical trials and the development of more selective, bioavailable PCD modulators to bridge this translational gap.
